# Supplementary material for: Are carbon emissions trading and green financial instruments synergistic? -Comprehensive quantitative research based on content analysis
Source: PLoS One. 2024 Mar 7;19(3):e0298601. doi: 10.1371/journal.pone.0298601 (PMC10919601; doi:10.1371/journal.pone.0298601)
Supplement: S1 Data — (ZIP) [file pone.0298601.s001.zip › Data/地级市Z_F工作报告.zip!/║ú─╧/║ú┐┌╩╨/└┤╫╘╣1⁄2╓┌║┼í╛┬φ┐╦╩2╛▌═°í┐_2006.docx]

　各位代表：

　　现在，我代表海口市人民政府向大会作《海口市国民经济和社会发展第十一个五年规划纲要的报告》，请各位代表连同《纲要(草案)》一并审议，并请市政协各位委员和其他列席人员提出意见。

　　一、“十五”时期经济社会发展回顾

　　“十五”时期，在省委、省政府的正确领导下，我市坚持以邓小平理论为指导，努力实践“三个代表”重要思想，树立和落实科学发展观，聚精会神搞建设，一心一意谋发展，经济社会发展取得显著成就，基本完成了“十五”计划任务。

　　(一)国民经济步入稳定快速发展轨道，综合经济实力显著增强

　　经济保持快速增长态势。全市生产总值突破300亿元大关，由2000年的175.23亿元增加到2005年的301.35亿元，年均增长12.1%;人均生产总值由12471元增加到17418元，年均增长8.6%。财政总收入突破60亿元大关，由2000年的23.1亿元增加到2005年的60.83亿元，增长了一倍多，年均增长21.4%。全社会固定资产投资由2000年的77.18亿元增加到2005年的137.17亿元，年均增长12.2%。产业结构进一步优化，三次产业结构从2000年的8.8：24.7：66.5调整到2005年的7.7：27.6：64.7。整体经济跃上一个新的台阶，综合经济实力和竞争力显著增强。

　　特色优势产业发展加快。工业化进程步伐加快。汽车工业实现跨越式发展，“药谷”建设快速推进，资源加工型产业持续增长，电子信息等新兴产业开始崛起，形成了以机械制造、医药、饮料食品、化学纤维业为主的产业结构。三星光缆、16万吨瓶级聚酯切片等一批项目建成投产，启动狮子岭飞地工业园区建设。2005年全市工业总产值达262.21亿元，五年年均增长22.8%。农业经济稳步发展。加大农业资金投入，加快调整优化农业结构，城郊型、服务型农业得到巩固和发展，无疫区建设取得成效，农业产业化发展加快，综合生产能力明显增强。2005年农业总产值达38.5亿元，五年年均增长7.6%。旅游业加速发展。石山火山群国家地质公园、海南热带野生动植物园等一大批景区、景点相继建成，凯莱、喜来登等一批五星级国际著名大酒店顺利开业，启动打造海口“娱乐之都”，成功举办旅游商品展销会、海南岛欢乐节、冼夫人文化节等系列旅游主题活动，积极组织参加海内外旅游促销活动，旅游业取得较好经济效益。2005年全市接待过夜游客512.2万人次，五年年均增长9.5%;旅游总收入47.2亿元，五年年均增长12.6%。商贸业繁荣活跃。完成了全市商业网点的规划建设，五年全市新增大中型商业网点121个，新增面积74.35万平方米。积极培育连锁经营、集中配送、电子商务等现代物流业，明珠广场、第一摩尔广场等一批商城建成运营，家乐福、国美等一批国内外著名零售企业签约落户，大大提升了海口的商业品位。2005年社会消费品零售总额138.45亿元，五年年均增长12.9%。

　　(二)城市建设步伐加快，人居环境明显改善

　　城市规划建设取得重大进展。创新城市规划理念，在全国省会城市首次进行了城市总体规划概念国际招标、总体规划纲要国内招标及专项规划设计国际招标。修编完成《海口市城市总体规划》。成立海口市城市规划委员会。启动城市规划四级管理机制。五年累计市政基础建设投资40.45亿元，比“九五”期间增加近12亿元，完成道路建设投资27.81亿元，新建、改造道路44条，总长126公里，面积570万平方米，龙昆南路、世纪大桥、滨海大道、国兴大道、秀英大道等建成通车，货运大道和绕城公路开工建设，美兰机场二期扩建投入运营，粤海铁路建成开通，区域交通枢纽地位进一步增强。自然村通车率99.2%，基本实现村村通公路的目标。城市燃气普及率96.8%，管道燃气普及率35%。供水、供电能力和港口吞吐量、信息通讯水平都有新的提高，城市服务功能不断增强。城市化步伐加快，2005年城市化率达59.5%，比2000年提高8.9个百分点。“十五”时期是全市城乡建设投入最多、变化最显著的五年。

　　人居环境建设成效显著。抓好美舍河、南渡江、东西湖等河、湖、渠水体整治改造，启动龙塘饮用水源保护工程，加强对演丰镇和三江镇湿地、东寨港国家自然保护区等生态脆弱区的保护。开展城市环境综合整治，五年拆除违法建筑62万平方米。停缓建工程处置取得重大进展，243宗、788万平方米停缓建工程复工建设，占总量的58%，收回闲置土地约4万亩，建成一批精品小区，房地产业健康发展。建成世纪广场、美舍河带状公园、华海花园及一批街心公园和绿地，新增改造绿地面积1047公顷，建成区绿地率达33.5%，森林覆盖率35%，绿化覆盖率达39.5%，人均公共绿地8.3平方米。重点工业污染源得到有效治理，城市空气质量继续保持国家Ⅰ级水平，先后荣获“国家环境保护模范城市”、“国家园林城市”、“中国人居环境奖”和“全国创建文明城市工作先进城市”等荣誉称号，城市品位进一步提升。

　　(三)改革开放步伐加快，发展活力进一步增强

　　各项改革顺利推进。全面启动政府机构和事业单位机构改革，加快理顺市、区两级管理体制，审批制度改革取得明显成效，行政审批事项大幅度减少，政府职能进一步转变。积极推行部门预算改革和国库集中支付改革。建立市级国有资产监督管理机构，加大对企业的监管力度，以粮食、商贸等国有企业为重点的产权制度改革基本完成。大力扶持个体私营等非公有制经济和中小企业发展，非公有制经济的活力和竞争力大大增强。建立“政府入口、市场出口”的城市建设投融资体制的基本框架，2005年与国家开发银行签订200亿元的开发性金融合作协议。加大城市经营力度，完成出租车、公交线路、广告经营权等市场化运作，美丽沙土地成功拍卖18.08亿元。

　　对外开放取得新成效。积极参与泛珠三角、大特区大都市“双大互动”等区域合作，不断创新招商引资方式，大力改善投资环境，鼓励企业积极参与国内外市场竞争，招商引资和对外贸易取得新进展，对外开放和国际交流不断扩大，韩国三星等6家世界500强企业相继进入海口。五年累计实际利用外资19.8亿美元。2005年全市外贸口岸进出口总额12.35亿美元，比2000年增长1.5倍，五年年均增长19.7%。

　　(四)社会事业加快发展，和谐海口建设全面启动

　　科技教育等各项社会事业取得新发展。大力实施“科教兴市”战略，培育高新技术企业109家，建立科技研发中心38个、技术创新示范企业14家，国家863生物医药成果产业化基地挂牌运作，取得科技成果429项，获得专利1171件，2005年高新技术产值达158亿元，比2000年增长4.5倍。教育事业快速发展，“两基”达标成果不断巩固提高，素质教育加快实施，职业教育快速推进，社会办学迅速发展，教育布局调整力度加大，城市教育综合改革稳步推进，普教经费总投入达30.78亿元，是“九五”时期的2.86倍。大力实施“文化精品”战略，歌舞剧《达达瑟》荣获第十届“文华奖”，广播剧《请到天涯海角来》、《西沙女兵》荣获“五个一工程”奖，首部《海口市志》出版发行。群众体育蓬勃发展，全民健身活动不断深入，竞技体育取得突破，在洲际体育赛事上共获得7金7银的好成绩，在全国体育赛事上共获得17枚金牌、24枚银牌、14枚铜牌。加强卫生基础设施建设，全市医疗卫生条件明显改善，医疗技术水平不断提高，公共卫生应急网络和公共卫生体系建设不断加强和完善，疾病预防工作取得重大成绩，农村与社区卫生工作不断加强，新型农村合作医疗试点工作取得明显成效，农民参合率达到76.3%。双拥工作成绩显著，连续五次荣获“全国双拥模范城”称号。民族团结进步事业成绩突出，2005年被国务院评为“全国民族团结进步模范集体”。妇女儿童工作成效显著，获“全国妇女儿童权益贡献奖”。计生、统计、审计、外事、侨务、民族宗教、人防、气象、档案等各项工作都取得新的成绩。

　　人民生活水平显著提高。2005年城市居民人均可支配收入9740元，比2000年增加2637元，年均增长6.5%，扭转了“九五”时期收入下降趋势。全面取消农业特产税，实行良种和农机具补贴等一系列政策，2005年农民人均纯收入3829元，比2000年增加976元，年均增长6.1%，比“九五”时期提高3.46个百分点。

　　和谐海口建设开局良好。为民办实事扎实有效。千方百计拓宽就业渠道，五年累计新增就业岗位9.9万个，国有企业下岗失业人员再就业2.8万人，转移农村劳动力2.9万人，城镇登记失业率控制在4%以内。积极实施农村饮水工程、城市路巷改造工程，推行城乡医疗救助工作，缓解了群众饮水难、行路难、看病难等问题。社会保障体系逐步健全，城乡低保覆盖面不断扩大、标准不断提高，47万人次进入低保范围。落实维护社会稳定各项工作措施，实现了全市无影响社会稳定的重大政治事件、无造成群死群伤的重大治安案件和事故、无集体进京上访事件的三个“零”的工作目标。深入开展基层安全文明小区和无毒社区创建活动，集中开展打击“两抢一盗”和毒品犯罪专项斗争，群众的安全感普遍增强，社会治安形势明显好转。建立公共突发事件应急处理机制，颁布实施《海口市突发公共事件总体应急预案》，制定25个专项预案，提高处置紧急事件的应变能力。大力整顿和规范市场经济秩序，加强安全生产目标管理，确保正常的社会秩序和市民生命财产安全。

　　(五)民主法制和精神文明建设不断推进，行政效能明显提高

　　民主法制进一步发展。自觉接受人大的法律监督、工作监督和人民政协、民主党派的民主监督，做到重大决策事项事先征求人大、政协的意见。虚心接受舆论和群众的社会监督。大力推进民主科学决策，设立市政府法律顾问、科技顾问，健全重大事项报告制度、质询制度、常务会议制度、民主评议制度及责任追究制度。认真抓好“四·五”普法教育，依法治市取得成效。五年来，市政府及各部门共办理人大议案和建议905件，政协提案1345件，办理三级人大代表视察建议80余件，提请市人大审议法规议案17件。积极贯彻《行政许可法》，提请人大修改地方性法规6件，清理政府规章34件，清理行政许可事项303项。

　　精神文明建设再上新台阶。开展保持共产党员先进性教育活动，真正达到了“党员受教育、群众得实惠”的目标要求。积极开展爱国主义和民族精神教育，大力实施《公民道德建设实施纲要》，贯彻落实中央《关于加强未成年人教育的意见》，加强社会公德、职业道德、家庭美德教育，深入开展文明机关、文明社区、文明诚信企业等群众性创建活动，城市文明程度和市民整体素质不断提高，青少年德育教育工作得到中央文明委的肯定。文明生态村建设全面开展，农村面貌有了很大改观，建成文明生态村640个，占自然村总数的29.2%，获“省文明生态村建设工作示范市县(区)”称号。

　　政府自身建设得到加强。深入开展投资环境建设活动，做好机关优质服务，建立市长接待日制度，正式启用海口市政务服务中心，建立行政首长问责制，全力营造一个勤政为民的政务环境。组织监督专员和特邀监察员走访百家企业听取意见，开展万人评议政府机关问卷调查，有力地促进了政府机关效能建设，提升了政府的公信度。加强制度建设，再造政府流程，依法行政意识不断增强，行政执法水平明显提高。

　　各位代表!过去五年，是在省委、省政府正确领导下，市委、市政府带领全市人民不断解放思想、开拓进取、团结拼搏的五年;是我市各项改革顺利推进、城市发展活力明显增强的五年;是我市经济社会发展取得显著成效、城乡面貌发生巨大变化、人民群众得到更多实惠的五年。在此，我代表本届政府，向奋斗在各条战线的全市广大干部群众及外地来海口的建设者，向给予我们支持的人大代表、政协委员、各民主党派、工商联和社会各界人士，向中央和各省区市驻市单位，驻市解放军、武警官兵，向所有关心和支持海口发展的港澳台同胞、海外侨胞和国际友人表示崇高的敬意和衷心的感谢!

　　我们也清醒地看到，“十五”期间我市经济社会发展还存在许多的困难和问题：一是经济总量小，综合竞争力不强，与兄弟城市差距拉大。二是产业发展层次低，自主创新能力弱，核心竞争力尚未形成。三是农村发展滞后，农民增收不稳定，镇域经济活力不足。四是中心城市聚集辐射能力差，城市特色不突出。五是就业压力较大，城乡居民收入不高。六是社会事业发展相对滞后，与城乡居民生活需求差距较大。七是制度性的障碍依然存在，体制和观念都亟需创新;投资环境建设的任务依然繁重，政府的行政效能有待进一步提高。对此，我们要高度重视，采取措施，认真加以解决，为我市“十一五”发展创造更加有利的条件。

　　二、“十一五”时期的奋斗目标和主要任务

　　“十一五”时期，是我市全面建设小康社会的关键时期，也是实现经济社会跨越式发展的重要机遇期。当前，国际形势有利于合作和发展，我国经济发展已步入结构升级、增长方式转变的新阶段，我省通过坚持不懈地夯实基础和优化环境，正在形成新一轮开发热潮。基于对当前形势的客观把握，我市认真贯彻中央、省关于“十一五”经济社会发展的指导方针和市委十届六次全会审议通过的《中共海口市委关于国民经济和社会发展第十一个五年规划的建议》，在深入调查研究、加强科学论证、广泛征求意见和反复推敲修改的基础上，本着立足我市实际、符合科学发展观和具有较强操作性的总体要求，着重体现加快经济发展、打造城市特色、构建和谐社会与提高创新能力，编制了《海口市国民经济和社会发展第十一个五年规划纲要(草案)》。

　　“十一五”规划，是我市全面贯彻落实科学发展观的第一个五年规划，《纲要(草案)》提出要把握好以下原则：必须以科学发展观统领全局，促进经济社会协调发展;必须加快转变经济增长方式，提高自主创新能力;必须大力调整经济结构，发展壮大特色优势产业;必须加快城乡一体化进程和生态城市建设，促进城乡区域协调发展和人与自然和谐相处;必须深化改革，扩大开放，增创体制新优势;必须坚持物质文明、政治文明、精神文明和社会建设四位一体，努力构建和谐海口。

　　《纲要(草案)》确定了“十一五”时期我市经济社会发展的主要目标：综合经济实力明显增强、社会事业全面发展、人民生活水平显著提高、城市功能进一步完善、改革开放深入推进、构建和谐海口取得新的进步。在实现“六大目标”的过程中，逐步形成产业特色、文化特色、城市特色、体制特色、环境特色，实现“三个翻番、两个突破”，即以2000年为基数，确保2010年生产总值、人均生产总值翻一番以上，力争城乡居民收入水平翻一番，生产总值突破500亿元，财政收入突破100亿元。建设宽裕型小康社会，努力跨入全国先进城市行列。

　　为此，必须全力抓好以下几个方面的重要工作。

　　(一)建设“四地一中心”，把海口发展成为辐射带动全省的中心城市

　　从提高产业层次和技术水平、提高规模和集聚效应入手，进一步调整产业结构，加快工业化进程，加快发展生物产业、现代服务业和都市型农业，壮大特色优势产业，不断提高经济发展的核心竞争力，落实省委、省政府提出的要把海口建设成为辐射带动全省的中心城市的要求。

　　建设全省以高新技术产业为导向的新型工业基地。坚持不污染环境、不破坏资源、不搞低水平重复建设的“三不原则”，加快发展现代制造业，大力推进工业化，重点发展壮大汽车制造、制药、化纤纺织、食品饮料、农产品加工等支柱产业，培育电子信息、精细化工、橡胶化工、旅游工业、生态环保等潜在优势产业。促进重点产业和新建项目集中发展，着力建设好保税区、高新技术产业开发区、海口“药谷”和汽车工业园等高端产业功能区。抓好狮子岭飞地工业园建设。加快发展节能型工业和资源再利用型工业，全面推行清洁生产，发展循环经济，把我市建成全省以高新技术产业为导向的新型工业基地。到2010年，全市工业增加值达到120亿元，占全市生产总值的23.6%。

　　建设全国热带滨海旅游休闲胜地。全面实施《海口市旅游产业发展规划》，积极推进“娱乐海口”工程，立足省会城市功能定位，实现以观光旅游为重点向以度假休闲旅游为重点转变。加大富有地方特色和特定文化内涵的旅游资源开发，突出旅游产品特色，重点规划建设一批文化娱乐项目和设施，打造滨海度假旅游休闲基地，抓好石山火山群国家地质公园、红树林生态旅游区等一批项目建设，努力开发一批旅游精品景点。加强旅游与其他产业的协调和融合，发挥省会城市综合优势，加强旅游区域协作，积极参与海口旅游圈旅游资源整合，把海口发展成为集观光游览、度假休闲、疗养健身、商务会展、文化娱乐为一体的多功能、综合型全国热带滨海旅游休闲胜地。到2010年，全市旅游总收入达到76亿元，年均增长10%。

　　建设全省都市型农业基地。坚持“富裕农民、服务城市”的方针，突出“无疫区、无公害”特色，充分利用城市提供的市场和生产技术条件，调整优化农业结构与布局，重点发展高效生态农业和观光休闲农业，为城市提供名、特、鲜、新农产品，为城市居民提供优美的生态环境。立足环境优势，建设沿海地区、美兰机场周边地区、东部南部地区和西部羊山地区特色产业基地，增强农业集聚效应。不断推进农业标准化生产，积极引进和推广新品种、新技术，在郊区建设1—2个高科技农业示范区，促进产业化经营和规模化发展，加速传统城郊农业向现代都市农业转变。以农产品出口为主攻方向，建设一批标准化的种植、养殖、加工出口示范基地，积极开拓国内外市场。到2010年，全市农业增加值达到32亿元，年均增长7%;畜牧业产值占农业总产值的50%。

　　建设国家海岛生物产业基地。紧紧抓住国内外生物科技革命和产业革命的重大战略机遇，充分发挥海南热带资源、生物海洋资源优势，加强技术开发，重点发展生物医药、生物农业、生物海洋产业，为建设国家海岛生物产业基地打下基础。力争到2010年生物医药产业增加值占工业增加值的20%。

　　建设区域交通枢纽和现代服务中心。依托我市空港、海港、火车站和汽车站“四位一体”等有利条件，完善海、陆、空交通功能和信息港功能，形成以海口为中心、覆盖全岛、畅通便捷的交通网和信息网，强化海口交通、信息枢纽地位。建设海、陆、空港物流园区，健全现代物流配送系统，形成现代化区域物流中心。实施《海口市商业网点规划》，建设市、区、镇商业中心，优化网点布局，完善配套设施，营造便利消费和物畅其流的消费环境;发展和壮大商业龙头企业，创新现代流通组织形式和服务方式，健全城乡商贸体系。加快发展现代物流、房地产、金融、中介服务和社区服务等服务业，不断增强海口区域性现代服务中心功能。到2010年，全市社会消费品零售总额达到244亿元，年均增长12%。

　　(二)提升城市品位，打造温馨宜居城市和理想的第二居住地

　　围绕打造城市特色，着眼于进一步增强城市集聚辐射功能，加快重大基础设施建设，基本建成现代化城市基础设施体系;着力于建立健全长效管理机制，不断提升城市管理的现代化水平;积极实施城市经营，推动城市资源优化配置;倡导环境文明和生态文明，加快环境友好型城市建设。到2010年，城市化率达到65%以上。突出城市特色。根据《海口市城市总体规划》，完善和实施分区规划、详细规划和各专项规划。组织编制和实施长流组团、江东组团建设规划，大英山城市中心区、海甸岛、新埠岛等片区开发规划，旧城和“城中村”改造规划。制定和实施区域合作发展规划，将周边地区纳入我市经济社会发展协调区，统筹区域产业发展、交通设施建设和生态环境建设。通过规划的实施，突出热带海岛、绿色生态、南洋建筑风格和温馨宜居城市特色。

　　完善城市功能。按照《海口市城市总体规划》要求，实施“温馨和谐海口”的八大工程，推进城乡建设。围绕产业发展，抓好一批关键性基础设施、公共服务设施及重要带动项目建设，引导产业集聚，加快完善我市产业发展的基础设施。加强城区道路网络建设，改造和平桥等三座桥梁，新建海新大桥等三座桥梁，形成以主干道为骨架，次干道、支线路合理分布的城市道路网络。着力抓好以能源供应、水资源保障、信息通讯、环境保护和城市安全为主体的基础设施建设，维护城市安全高效运行。加强文化、体育、医疗、卫生等社会事业基础设施建设，提高人民群众的生活质量。

　　优化城市环境。发展社区服务和社会公益事业，推进和谐社区、和谐村镇建设，培育城市宜居文化。“十一五”时期，计划安排城市环境建设保护资金20亿元，占政府投资的6.7%，是“十五”时期的2.7倍。落实《海口生态市建设总体规划》，以对水源、绿地、森林、海洋、湖泊、河流等自然资源的生态保护和建设为重点，加强生态环境建设;强化对城市水系的综合治理，完成美舍河、五源河、龙昆沟、大同沟、东西湖、红城湖的治理，构筑城市活水系，形成江、海、湖、河贯通的“水城”;继续推进“三边林”建设，实施城乡大环境绿化建设，形成林带环绕、绿树成荫的“林城”;科学规划和配置城区花草种植，新建10个公园和100个街边绿地、小游园，促进城乡环境美化，形成鲜花盛开、姹紫嫣红的“花城”，不断扩大城市绿色空间，力争2010年森林覆盖率达39%，城镇人均公共绿地面积12平方米。

　　强化城市管理。要统筹规划、建设与管理，把管理放在更加突出的位置。健全完善城市管理法规，形成较为完整的城市管理法规体系，推进城市依法管理。科学划分市、区、街道、居委会的事权界限，加强区、街道、居委会的管理能力建设，充分发挥区、街在城市管理中的主体作用和人民群众的参与作用。认真落实城市管理权限属地化的各项政策，完善城管执法目标管理、执法保障体系和公共城管联动机制建设，积极推进城管执法工作向中心镇延伸。继续推进违法建筑、交通秩序、市容市貌、市场秩序、安全生产等方面的城市环境综合整治工作，使城市面貌发生实质性变化。深化城市管理体制改革，推进城市管理综合执法，健全法治、科学、长效的管理机制，提高城市管理整体水平。

　　拓展城市经营。整合、重组城市各种优势资源，运用市场机制经营城市，重点加强城市资源的包装营销，积极推进城市资源资本化，鼓励各种资本进入基础设施建设领域，促进城市资源增值，实现城市资源优化配置。继续抓好土地规划、整理、储备和经营开发，加强土地资源成片推介，通过策划包装推向市场。拓展旅游房地产市场，积极培育旅游房地产业。推进城市基础设施的市场化运作，城市供水、污水处理、公交、环卫、园林绿化等逐步实现社会化投入、市场化经营和企业化管理。充分挖掘城市无形资产，为城市可持续发展筹集更多资金。

　　(三)统筹城乡发展，建设社会主义新农村

　　加强城乡互动，改善农村生产生活条件，发展农村经济，增加农民收入，提高农业综合生产能力。以文明生态村为综合创建载体，建设“生产发展、生活宽裕、乡风文明、村容整洁、管理民主”的社会主义新农村。“十一五”时期，计划安排“三农”资金18亿元，占政府投资的6%，是“十五”时期的4.9倍。

　　加强城乡互动。探索建立城乡统筹的新机制，统筹城乡经济社会发展，建立“工业反哺农业、城市支持农村”的长效机制，运用工业化理念和规模经营方式，全面提高农业综合生产能力。加强部门间的统筹协调，形成支持农村发展的合力。坚持“多予少取放活”的方针，继续完善和强化各项支农政策，完善扶持农业工作机制，加大对农村投资的倾斜力度，尤其要加大对贫困乡村发展的支持力度，扩大公共财政覆盖农村的范围，让全市农民共享改革发展的成果。

　　发展镇域经济。发展镇域经济是加快我市城乡一体化的重要载体，制定和实施《海口市加快镇域经济发展的决定》及《海口市扶持镇域经济发展的若干规定》，加强城镇规划编制工作，完成全市23个小城镇的规划编制，重点完善10个中心镇基础设施和公共服务设施;制定镇域产业发展规划，建设各类专门性市场，加快家具、花卉、建材、服装等部分专门性市场向近郊城镇转移。根据镇、村的不同特点，因地制宜，培育发展优势产业，不断提高镇域经济整体实力，促进人口向小城镇转移。

　　致力增加农民收入。健全农民增收长效机制，坚持比较效益最大化和生态平衡原则，以市场为导向，加快农业产业化进程，积极发展农村第二、第三产业，加快农村经济结构调整步伐，拓宽增收渠道。开展“10万农民大培训”活动，加强对农民的劳动就业技能培训，提高农民就业能力。继续建立农村劳务市场，建立和完善农民工劳动合同制度，做好农民外出务工就业的组织、指导和服务工作，鼓励和支持农民工异地劳务输出，组织更多农民工到珠三角等国内发达地区打工。5年转移农村劳动力6万人。到2010年，力争农民人均纯收入5700元，年均增长8%左右。

　　改善农村生产生活条件。健全财政支农资金稳定增长机制，扩大公共财政覆盖农村的范围，创新政府支农资金管理方式，同时，加大对农业的投入。落实我市制定的鼓励投资农业政策，全方位拓展农业投资渠道，积极引进社会资金发展农业。做好中型和小(一)型水库除险加固工程、水库灌区防渗配套工程，加强水库工程建设，完成千亩以上田洋整治任务，实施羊山地区农业用水工程，解决羊山地区农业缺水问题，推进农田水利基础设施建设。加快农村道路硬化、厕所改造、清洁能源使用和禽畜圈养，推行新的垃圾收集处理模式，建设适合乡村的排水和污水处理设施，继续推进农村路通、水通、电通、信息通工程建设，缩小城乡生活条件的差别。加快农村教育、卫生、文化、科技等社会事业发展，整体改善农村居民的生产生活条件，让农村居民更多地享受经济社会发展的成果。

　　切实保障农民权益。认真贯彻实施农村土地承包法，建立规范的土地使用权流转机制，完善对征地农民的合理补偿机制和失地农民基本生活保障制度，治理农村土地承包纠纷问题。深化农村体制改革，抓好村镇机构及农村税费配套改革，进一步减轻农民负担;推动生产组织形式和经营模式创新，支持农村各类合作组织发展。推进农村民主管理，建立健全村民委员会民主选举和民主议事、民主监督制度，推行村务公开，确保广大农民依法行使当家作主的权利。

　　建设文明生态村。建立健全“以城带乡、城乡共建”的创建机制，深入开展干群共建、工农共建、村企共建、军(警)民共建和村校共建等活动，全力推进文明生态村创建，到2010年，全市65%以上的自然村建成文明生态村。要把创建文明生态村工作与农村工作、扶贫工作、文教卫生、农业产业调整、农村水电网改造、科技兴农、社会治安综合治理、计划生育、思想道德建设等工作密切结合起来，开展综合创建;统筹城乡规划，将规划管理延伸到村镇;加强文明生态集镇建设，形成文明生态村和文明生态集镇共同进步、协调发展的新局面。

　　(四)推进改革开放，营造一流的发展环境

　　“十一五”期间，继续推进政府职能转变，基本完成国有企业改革，加快发展民营经济，分类推进社会事业管理体制改革，深化投融资体制和财政体制改革，完善社会主义市场经济体制，提高对外开放水平，努力形成非国有企业为主体、市场调节为主、社会自我管理水平较高、政府廉洁高效、外向开放的海口体制特色。

　　加快行政管理体制改革，进一步改善投资软环境。以转变政府职能为重点，继续推进行政管理体制改革，减少和规范行政审批，切实把政府职能转到经济调节、市场监管、社会管理和公共服务上，提高社会管理和公共服务水平。调整和完善政府机构设置，明确职能分工，理顺市与区的权责关系，合理划分权限。加强行政法制建设，建立权责明确、行为规范的行政执法体制，严格按照法定权限和程序行使职权、履行职责，认真落实行政执法责任制和行政执法过错追究制度，完善和执行行政首长问责制等制度，提高政府依法行政水平和行政效能。创新政府行政方式、方法，加快电子政务建设，推行政务信息公开，扩大网上办公范围，促进政府部门资源共享。完善行政管理决策机制，坚持实行民主集中制，加强专家咨询、社会公示和听证、决策执行评估等制度建设。

　　深化国有资产管理体制改革，促进非公有制经济发展。加快市属国有企业的改制重组和股权调整，基本完成国有企业改革。以旅游、水务、公交、城建领域为重点，加快优势资源和资产整合，组建水务、公交、城建集团公司，进一步完善国有资产监督管理体系，加强对国有资产运营监管，确保国有资产保值增值。完善和落实扶持非公有制经济发展的各项政策措施，创造平等竞争、一视同仁的法制、政策和市场环境，保护非公有制企业的合法权益，提高和扩大非公有制经济发展水平和规模。放宽准入标准，鼓励非公有资本进入基础设施、基础产业、公用事业领域，参与国有企业改制和重组。建立和完善社会化服务体系和信用担保体系，充分发挥中小企业融资担保公司作用，切实帮助企业解决融资问题。

　　深化财政管理体制和投融资体制改革。合理调整财政支出，提高对公共安全体系、公共卫生体系等社会事业的支出比重。按照事权和财权相统一的原则，完善市区财政体制，明确两级政府的支出责任和范围。加强财政资金使用管理，提高资金使用效益。强化部门预算，全面推行国库集中支付，加强政府采购和收支两条线管理。落实企业投资自主权，对非政府投资的建设项目，不再实行审批制，推行社会投资备案制和核准制。规范政府投资行为，增强政府投资的导向和带动作用，对非经营性政府投资项目建设推行工程总承包制度，加强审计监督，完善政府投资监督评价机制。

　　加快社会事业管理体制改革。创新社会事业管理模式，合理界定经营性和非经营性社会事业项目，建立社会事业建设和管理的多元投资机制，鼓励和吸引社会投资进入教育、文化、体育、卫生等领域。继续推进教育体制改革，形成以政府办学为主体，公办学校与民办学校共同发展的格局。深化文体体制改革，加快职能转变，把注重部门管理转到行业管理上来，把注重于“办文化、体育”转到“管文化、体育”上来，推进文化行政综合执法体系建设。继续推进医疗卫生体制改革，着力解决群众看病难、看病贵问题。

　　提高对外开放和区域合作水平。以园区招商、产业招商和项目招商为重点，积极引进大企业、大集团来海口投资建设。抓好项目的筛选储备和推介，加强对已签订协议及在建项目的跟踪服务，促进项目开工建设，全面提高招商引资的质量和水平。坚持实施“走出去”战略，积极参与区域经济合作，进一步加强与“泛珠三角”等地区和东盟各国的经贸交流与合作，推动与琼北周边地区的资源整合，促进区域协调发展。“十一五”期间，外商直接投资年均增长10%，外贸出口总额年均增长12%。

　　(五)统筹社会发展，提高覆盖城乡的公共服务水平

　　统筹经济社会发展，统筹城乡发展，加快公共资源向社会事业、向农村倾斜，提供覆盖城乡的公共服务，促进均衡发展。“十一五”时期，计划安排资金65.5亿元用于发展社会事业，占政府投资的21.8%，是“十五”时期的1.7倍。

　　提高科技创新能力。把自主创新能力建设摆到更加突出的位置，大力实施科教兴市、人才强市战略，建立以企业为主体、市场为导向、产学研相结合的技术创新体系;围绕主导产业，帮助一批骨干企业建立研发机构，增强企业科技创新能力，培育和拥有更多的自主知识产权。通过建设维堤瑷生物研究院、海洋生物技术研发中心等机构，推动生物医药产业化基地、海洋生物技术研发基地的建设，大力开发和推广应用新技术、新产品，加快科技成果向现实生产力转化，大力发展高新技术产业。加快科技服务体系建设，设立生产力促进中心，抓好农业科技服务。加快完善培养人才、聚集人才、激励人才的工作机制，为经济社会发展提供强有力的人才保障和智力支持。加强知识产权保护，建立健全区域科技创新体系，为构建创新型城市提供强有力的科技支撑。

　　优先发展教育事业。全力推进中小学标准化建设，优化教育环境，到2010年小学、初中、高中优质学位率分别达到35%、55%和75%。重视发展农村教育，调整农村中小学布局，完成危房改造。全面落实“两免一补”政策，全部免收九年义务教育杂费和课本费，坚持对困难家庭学生给予寄宿生活费补助。着力提高教师整体素质，实施城乡教师交流制度，促进义务教育均衡发展。基本普及高中教育，积极发展成人教育。推动中职和高职教育协调发展，到2010年职业学校增加15所，在校生总数达到3万人。加快发展高等教育，争取成建制引进三到五所高校，在海口市职业经济技术学院的基础上创办海口大学，到2010年市属各类高等教育在校生达6万人。推进学习型城市建设，初步形成现代国民教育体系和终身教育体系。

　　加快发展文体事业。加快文体基础设施的建设，积极发展公益性文化事业，满足人民群众日益增长的文化生活需求。依法做好历史文化名城的保护工作，充分挖掘、保护、利用文化资源，打造独具特色的文化品牌。全面建设公共文化服务体系，重视发展社区和农村公共文化事业，丰富基层文化生活。大力发展广播电视、新闻出版事业，进一步扩大广播、电视人口覆盖率。强化文化市场和互联网管理，净化青少年成长环境。深入开展全民健身活动，提高竞技体育水平。积极推进足球冬训、高尔夫球、帆船训练、潜水培训等项目建设，加快文化、体育产业发展，努力建设文化海口。

　　大力发展医疗卫生事业。做好预防保健和重大疾病、传染病防治工作，加强疾病预防控制、医疗救治和卫生执法监督，全面提高医疗和公共卫生服务水平。加快发展社区医疗卫生事业，实施社区卫生服务机构标准化建设。改善农村医疗卫生条件，搞好中心镇卫生院建设，每个行政村设立1个以上医疗服务点，完善巩固农村新型合作医疗制度。整顿和规范医疗市场，严厉打击非法行医、制售假冒伪劣产品违法行为。加强妇幼保健工作，深入开展爱国卫生运动，普及健康教育，建设健康城市，到2010年力争城市居民健康水平和卫生服务指标达到国内先进水平。

　　(六)坚持以人为本，构建和谐社会

　　着眼于解决人民群众最关心、最直接、最现实的利益问题，促进社会充分就业，提供社会保障，加强精神文明和民主法制建设，维护社会稳定和公共安全，着力推进和谐海口建设。

　　努力扩大社会就业。认真落实积极的就业政策，重视发展就业容量大的服务业和劳动密集型产业，开发更多的就业岗位。落实财政补助、小额贷款和税费减免等优惠政策，做好下岗职工、大中专毕业生和复转军人安置工作，完善对困难群众的就业帮扶援助机制。加快劳动力市场建设，建立市场导向的就业机制。加强劳动技能培训，建立政府扶助、社会参与的职业技能培训机制，建立覆盖城乡的就业服务体系，促进城镇失业人员再就业。“十一五”期间，全社会从业人员年均增加3.2万人，城镇登记失业率控制在4%以内。

　　完善社会保障体系。增加财政对社会保障的投入，多渠道筹措社会保障资金，扩大社会保障覆盖面。加强社会保险基金征缴和管理，确保各项保险金依法按时足额支付。健全医疗、失业、工伤、生育保险制度，加快基本养老金计发办法改革，规范企事业单位退休人员社会化管理。完善农村养老保险制度，解决被征地农民的社会保险问题。完善社会救助体系，提高最低生活保障标准，保障城乡困难群众的基本生活，逐步形成覆盖城乡、全市统一、多层次的社会保障体系。充分发挥社会慈善、社会捐赠、群众互助在扶贫帮困中的作用，推动慈善事业和社会福利事业发展。

　　加强社会主义精神文明建设。贯彻落实《公民道德建设实施纲要》、《关于加强未成年人教育的意见》，加强社会公德、职业道德和家庭美德教育，增强市民法律意识、公德意识，加快社会主义思想道德体系建设。倡导科学、文明、健康的生活方式，大力弘扬遵纪守法、诚实守信的社会风尚，培育和发扬市民的自主创业意识和爱岗敬业精神，普及科学文化知识，提高全民素质。广泛开展群众性精神文明创建活动，加快文明城市、文明社区、文明村镇、文明行业建设，提升城乡文明程度，争创全国文明城市。

　　加强民主法制建设。坚持依法行政，自觉接受人大和社会各界监督，认真听取政协和民主党派、工商联、无党派人士的意见，广泛征集专家学者和市民建议。认真办理人大代表的议案、建议和政协委员的提案，提高办理的质量和效率。充分发挥工会、共青团、妇联等人民团体的桥梁和纽带作用。深入实施政务公开，推广厂务公开。扩大基层民主，尊重基层和群众的民主权利，完善城乡居民自治。加强政府立法工作，强化行政执法监督。开展“五·五”普法教育，在全社会营造学法、懂法、守法的良好氛围。

　　加强公共安全建设。高度重视公共安全工作，全力维护人民生命财产安全和维护正常社会秩序。加强预防、避险、自救、互救、减灾等知识宣传教育，提高社会公众应对公共安全突发事件能力。加强台风、洪涝、地震、火灾和交通、食品安全、公共卫生事故等重大公共安全突发事件应急体系建设，建立健全预测预警机制、响应处置机制及应急保障机制，增强政府有效处置公共突发事件的能力。

　　努力保持社会稳定。完善和落实社会治安综合治理各项措施，推进社会治安防控体系建设。坚持“严打”方针，严厉打击黑恶势力、贩卖毒品等严重违法犯罪活动。加强情报信息工作，建立有效预警机制，提高预防、发现和控制各类违法犯罪的能力。做好预防和减少青少年犯罪工作。坚决扫除各种社会丑恶现象。重视做好人民群众来信来访工作，加大矛盾排查、调处力度，维护社会稳定。关心和支持部队建设，加强国防教育，推进双拥共建工作，巩固和发展军政、军民团结。做好人口和计划生育、外事、侨务、民族宗教、老龄、档案等工作，依法保障妇女、未成年人和残疾人的合法权益。

　　(七)实施“八大工程”，夯实经济社会发展的物质基础

　　“十一五”期间加快我市发展必须保持应有的投资力度，全市五年投资总额将达920亿元，年均增长10%，全面实施“八大工程”。

　　“便捷海口”工程。加强各类对外交通和通信设施建设，强化中心城市交通、通信枢纽功能。实施马村港、秀英港扩建工程，规划建设航空物流中心、客运中转基地，完成绕城公路建设，完善对外交通公路体系和城市道路网络，提高信息传输和服务功能，提升区域性海洋运输、航空运输、公路交通、信息港枢纽地位。

　　“品质海口”工程。提升城市品位，改变城市面貌，拓展城市空间。启动大英山城市中心区、美丽沙、新埠岛建设，加快长流新区开发，择机启动江东组团建设，加快推进旧城和“城中村”改造步伐，优先实施小街小巷改造、城市亮化等民心工程，合理布设大型公园、步行街、街心公园，增加绿色公共空间和公共设施，建设最佳人居环境。

　　“产业海口”工程。围绕产业发展进行市政基础设施配套，基本完成三大工业园区、保税出口加工物流园区和农产品会展中心的配套设施建设，建设沃尔玛购物广场等大型商业设施。进一步建设完善城市供水系统，加大供电工程建设力度，完成城乡电网改造，扩大燃气管网覆盖范围，为我市工业、现代物流业、商贸业的快速扩张及支柱产业的发展壮大创造条件。

　　“娱乐海口”工程。围绕打造“娱乐之都”，完善相关基础设施配套，加快旅游娱乐业发展，为游客、市民的吃、住、游、玩提供良好条件。重点规划建设一批度假酒店、酒吧街、购物街、休闲娱乐会所、游艇码头和高尔夫球场，在主要旅游区配套建设文化、体育、疗养设施，抓好西海岸体育休闲长廊等项目建设。

　　“人文海口”工程。提高公共服务和社会管理水平，抓好教育、医院、文体等社会事业基础设施的合理布局与建设，建立健全城乡医疗卫生体系，加快文体设施建设，完善城市社会服务功能。有计划、高标准实施历史文化名城保护工程，选择一批地方特色较突出、历史原貌保留较好的村镇，予以重点修整和保护，丰富海口民间民俗文化内涵。

　　“生态海口”工程。规划建设长流、江东污水处理厂和垃圾处理场项目，加快推进我市垃圾、污水处理产业化进程。全面启动“一园两湖”改造、红城湖整治工程，加大地表水水体整治和饮用水源保护力度，实施红树林生态恢复工程。完成美舍河等几条河系的治污，高标准地进行城市绿化美化，保护和优化生态环境。

　　“平安海口”工程。健全城市综合防灾减灾系统，完善城市安全保障体系，确保城市综合安全。建立和完善以110系统为基础，包括治安、消防、人防、三防、防震在内的统一的公共安全保障体系及指挥系统，提高对公共安全事故和自然灾害的应急处置能力。加强南渡江、新埠岛、海甸岛防洪、防潮工程建设，完善城市消防和人防工程建设。完成公共安全应急处置中心、强制戒毒所等政法系统基础设施建设，确保社会平安稳定。

　　“田园海口”工程。加快社会主义新农村建设步伐，促进城乡经济社会协调发展，实现都市风光与田园风光的完美结合。抓好10个中心镇规划建设。加大农村基础设施投入，加快水、电、路、通讯、广播电视“五网”建设，基本完成所有适宜推广沼气村镇的“一池三改”，实现人畜安全饮水目标，改善农村居民生活条件。加大农村教育的投入，改善农村医疗条件，建立农村医保，加快文明生态村建设。

　　三、全力以赴做好2006年各项工作，努力实现“十一五”良好开局

　　2006年是“十一五”规划的起步之年，做好全年的各项工作至关重要。2006年我市经济和社会发展的主要指标是：生产总值增长12%，财政总收入增长15%，全社会固定资产投资增长15%，社会消费品零售总额增长12%，城市居民人均可支配收入增长8%，农民人均纯收入增长6%，人口自然增长率控制在8‰以内，城镇登记失业率控制在4%以内。2006年政府的投资将向“三农”、民生和环保领域倾斜，安排在这三个领域的资金分别是2005年的3.8倍、1.7倍、3.1倍。为了实现2006年的发展目标，要着力抓好以下工作。

　　(一)确保经济平稳快速增长。要加快发展现代制造业、旅游休闲业、旅游房地产业、现代物流业，确保开局之年经济稳定增长。要加强部门沟通协调，多渠道筹集建设资金，抓好“八大工程”等投资项目的启动工作，强化项目工作责任制，确保2006年投资计划的顺利实现;完成海汽发动机项目的设备安装调试，抓好15个配套厂项目的按期入园，加快海马二期、药谷二期及狮子岭飞地工业园的基础设施建设，引导工业项目向园区集中，形成集聚效应和规模效益，力争实现工业总产值283亿元，工业增加值66亿元;完成《海口市旅游产业发展规划》，编制《海口市旅游资源目录》和《海口市在建拟建旅游项目库》，抓好石山火山群国家地质公园、东寨港红树林生态风景区、热带植物博览园等项目建设，加强城市营销力度，优化旅游环境，拓展旅游房地产市场，力争实现旅游总收入51亿元;调整商业布局，重点抓好国贸、老城区、府城和秀英小街等商业功能区建设，支持家乐福等按期运营，积极推进美兰机场、火车站、港口等物流园区建设，加快发展现代物流业，力争实现商品零售总额155亿元。

　　(二)确保社会主义新农村建设有良好开端。按照新农村建设的总体要求，以创建文明生态村为综合载体，以发展经济、增加农民收入为中心任务，扎实推进我市新农村建设。完成二十三个乡镇的建设规划，抓紧编制镇域产业发展规划，制定实施发展镇域经济的决定和扶持办法，加快城市功能向城郊镇转移。安排资金2450万元加快农田水利工程和安全饮水工程的建设，积极争取省政府的支持，落实资金4500万元推进农村公路“村村通”工程建设。继续打造无疫区品牌，建设19个种猪基地;推进标准化生产，建设4个标准化生态蔬菜示范基地;扶持农产品出口，建设畜禽生产出口基地;筹建全省最大的热带农产品展示中心;发展海洋捕捞、海淡水养殖和水产品加工;力争全年完成农业总产值41.2亿元，畜牧业总产值占农业总产值的40%，水产品总产量5.8万吨，加工出口2.5万吨。大力发展打工经济，启动建设农村劳动力培训基地，完成3万人次的农业技术培训和1万人次的农民劳务技能培训，全年力争转移输出农村劳动力5000人以上。进一步加大文明生态村建设力度，做到全面发动、整体推进，争取全年新建168个文明生态村，覆盖率达到35.6%。

　　(三)确保民生问题得到妥善解决。要以构建和谐社会为目标，下大力气解决群众关心、社会关注的就业、收入、住房、教育、医疗、社保等问题，切实为群众谋福利。千方百计扩大就业，全年力争新增就业岗位2.8万个，城镇登记失业率控制在4%以内。积极扶持弱势群体，进一步改善中低收入家庭住房条件，通过租金补贴、租金核减、提供廉租房等途径，缓解200户特困居民和100户农村残疾人以及城镇低收入者的住房困难问题;安排资金5800万元，用于完成农村学校危房改造和布局调整，免费为300名特困家庭子女提供市属职业教育、补贴困难家庭子女义务教育寄宿生活费;完成8个镇卫生院的改扩建任务，缓解农民看病难问题。推进城乡社会保障体系建设，安排2700万元资金，用于扩大城乡低保范围，力争城区居民和农村居民低保人数均达到5%;安排100万元在全省率先建立专项基金，着力解决进城农民工的工伤保险问题;安排资金1000万元，继续完善新型农村合作医疗制度，提高市级对参保农民的补助标准，力争全市农民参保率达80%以上。安排300万元，用于城镇无业居民社区卫生医疗服务，覆盖城区无业居民30万人。努力保障公共安全，高度重视安全生产，落实安全生产责任制，提高处置公共突发事件的能力，大力推进“平安海口”工程。全力办好20件直接关系群众生活的实事。

　　(四)确保城市形象进一步提升。以启动八大片区开发为重点，努力把海口打造成热带海岛、绿色生态、南洋建筑风格和温馨宜居的城市。提高城市品位。完成南渡江地带和东海岸城市设计招标方案，完成海甸溪北岸改造规划，组织编制控规，着手制定城市静态交通组织规划。加快城市建设步伐。推进货运大道、滨江西路、海口绕城公路的建设，实施大同路、海秀东路慢车道、琼州大道东半幅、大英后路、长堤路改造，继续完成城市次干道改造，打通断头路;落实资金3800万元加快改造小街小巷和安装路灯;推动海甸岛、新埠岛、长流新区、桂林洋大学园区、江东旅游区、大英山城市中心区等片区建设，实施“一园两湖”和“红城湖”的改造、整治，做好美丽沙片区开发的服务工作;加快城市公园、街心花园及公共绿地建设，全面推进城市绿化;建成一批垃圾转运处理配套设施。提高城市管理水平。认真落实《海口市环境污染行政责任追究办法》，坚决查处破坏环境的事件;抓好违章建筑、交通秩序、市容市貌、市场秩序、安全生产等专项整治，2006年力争清理并拆除违法建筑50万平方米。

　　(五)确保创新能力建设有明显起色。通过推进体制改革，加大技术创新，提高我市的整体创新能力。推进改革开放。完成椰树集团的改制、椰岛股份公司的股权分置改革及煤管总的股权调整，组建水务集团、公交集团、城建集团，做好港航控股公司的移交管理工作;贯彻实施《海口市市级事业单位机构改革实施方案》，积极推进事业单位机构改革和人事制度改革;大力推进政府管理创新和和制度创新，启动城市管理综合执法，推行“数字化、网络化、下沉式”城市管理体制;成立政府投资项目管理中心，规范重点项目决策制度;成立工业发展职能部门，强化工业发展带动战略;成立专门招商机构，统筹全市资源，理顺招商体制，组织本地企业到国内外进行产品推介、项目招商，精心策划好“海口市城市推介暨投资洽谈会”等重大招商项目，力争全年实现外商直接投资4.3亿美元。加大技术创新力度。将海口保税区、狮子岭飞地工业园等所有市属园区纳入海口高新技术园区范畴，形成“一区多园”的格局;整合科技要素，实现资源共享;增加科技三项经费投入、落实高新技术产业风险资金，提高我市高新技术自主创新能力;鼓励企业加大研发投入，努力开发一批事关我市经济社会发展和提升产业核心竞争力的关键技术。

　　加强政府自身建设，是全面完成2006年各项工作任务和实现“十一五”宏伟目标的重要保证。要高度重视人大代表、政协委员的意见、建议，自觉接受人大及其常委会的法律监督，自觉接受人民政协的民主监督，全面推行行政执法责任制，提高依法行政水平;要在提高经济管理水平的同时，更加注重社会管理，把财力、物力更多地放到推进社会发展和解决民生问题上来，把精力、注意力更多地用到促进社会公平和正义、维护社会秩序和稳定上来，加快建立公共危机防控体系和社会矛盾化解机制，不断增强处置突发事件的能力，提高公共服务水平;要按照省委《关于加强投资环境建设若干问题的决定》，认真落实市委加强经济发展环境建设的总体要求，加强政风建设，切实提高政府办事效率和服务水平，优化发展环境;贯彻实施《中华人民共和国公务员法》，进一步规范公务员管理，加强对公务员的监督，建设高素质的公务员队伍;严格贯彻《市直单位领导班子重大问题议事规则》，增强各单位一把手集体决策的意识，规范决策程序，提高决策水平;严格执行廉政建设各项规定，从严查处各种违纪违法案件，树立“为民、务实、清廉”的政府形象，努力建设人民满意政府。

　　各位代表，我市“十一五”发展蓝图经本次人民代表大会审定，将成为号召、凝聚全市人民团结奋斗的行动纲领。海口发展将进入新的重要历史时期，我们既感到任务艰巨，也充满信心。我们坚信，在全市人民的共同努力下，五年后海口将会成为一个充满热带海岛风情、环境优美舒适、生活质量一流的温馨宜居城市。让我们在新的历史起点上，更加紧密地团结在以胡锦涛同志为核心的党中央周围，高举邓小平理论和“三个代表”重要思想的伟大旗帜，以科学发展观统领全局，在市委的正确领导下，万众一心，艰苦奋斗，开拓创新，求真务实，共同创造海口更加美好、灿烂的明天!
